# Cardiovascular diseases in Africa in the twenty-first century: Gaps and priorities going forward

**DOI:** 10.3389/fcvm.2022.1008335

**Published:** 2022-11-10

**Authors:** Neema W. Minja, Doreen Nakagaayi, Twalib Aliku, Wanzhu Zhang, Isaac Ssinabulya, Juliet Nabaale, Willington Amutuhaire, Sarah R. de Loizaga, Emma Ndagire, Joselyn Rwebembera, Emmy Okello, James Kayima

**Affiliations:** ^1^Rheumatic Heart Disease Research Collaborative, Uganda Heart Institute, Kampala, Uganda; ^2^Kilimanjaro Clinical Research Institute (KCRI), Moshi, Tanzania; ^3^Department of Global Health, University of Washington, Seattle, WA, United States; ^4^Department of Adult Cardiology, Uganda Heart Institute, Kampala, Uganda; ^5^Heart Institute, Cincinnati Children’s Hospital Medical Center, Cincinnati, OH, United States; ^6^Department of Pediatric Cardiology, Uganda Heart Institute, Kampala, Uganda; ^7^Department of Medicine, Makerere University College of Health Sciences, Kampala, Uganda; ^8^Department of Medicine, Case Western Reserve University, Cleveland, OH, United States; ^9^Department of Pediatrics, University of Cincinnati, Cincinnati, OH, United States

**Keywords:** cardiovascular diseases, cardiovascular medicine, Africa, sub-Saharan Africa (SSA), priorities, gaps, SDG 3.4

## Abstract

In 2015, the United Nations set important targets to reduce premature cardiovascular disease (CVD) deaths by 33% by 2030. Africa disproportionately bears the brunt of CVD burden and has one of the highest risks of dying from non-communicable diseases (NCDs) worldwide. There is currently an epidemiological transition on the continent, where NCDs is projected to outpace communicable diseases within the current decade. Unchecked increases in CVD risk factors have contributed to the growing burden of three major CVDs—hypertension, cardiomyopathies, and atherosclerotic diseases- leading to devastating rates of stroke and heart failure. The highest age standardized disability-adjusted life years (DALYs) due to hypertensive heart disease (HHD) were recorded in Africa. The contributory causes of heart failure are changing—whilst HHD and cardiomyopathies still dominate, ischemic heart disease is rapidly becoming a significant contributor, whilst rheumatic heart disease (RHD) has shown a gradual decline. In a continent where health systems are traditionally geared toward addressing communicable diseases, several gaps exist to adequately meet the growing demand imposed by CVDs. Among these, high-quality research to inform interventions, underfunded health systems with high out-of-pocket costs, limited accessibility and affordability of essential medicines, CVD preventive services, and skill shortages. Overall, the African continent progress toward a third reduction in premature mortality come 2030 is lagging behind. More can be done in the arena of effective policy implementation for risk factor reduction and CVD prevention, increasing health financing and focusing on strengthening primary health care services for prevention and treatment of CVDs, whilst ensuring availability and affordability of quality medicines. Further, investing in systematic country data collection and research outputs will improve the accuracy of the burden of disease data and inform policy adoption on interventions. This review summarizes the current CVD burden, important gaps in cardiovascular medicine in Africa, and further highlights priority areas where efforts could be intensified in the next decade with potential to improve the current rate of progress toward achieving a 33% reduction in CVD mortality.

## Introduction

Approximately three decades ago, conditions such as hypertension and atherosclerotic heart diseases were rare in Africa, and communicable diseases were the major causes of death (1). Historically prioritized on the World Health Organization’s (WHO) agenda, efforts to curb infections such as HIV, tuberculosis and malaria have been fruitful with remarkable declines in the burden of communicable, maternal, neonatal and nutritional (CMNN) diseases since 2005 (2). Unfortunately, over this time, non-communicable diseases (NCDs), in particular cardiovascular diseases (CVDs), have shown an unparalleled rise. Surpassing HIV/AIDS, malaria and other enteric infections in the top 10 causes of death, CVD jumped from the 6th to the 2nd leading cause of death in sub-Saharan Africa (SSA) between 1990 and 2019 (2). The underlying drivers have been marked increases in major risk factors, such as hypertension and diabetes (3), risking to offset the substantial health gains made with communicable diseases. The current status is such that the age standardized DALYs due to CMNNs are almost at par with NCDs (4, 5). The epidemiological shift behind this trend has been heavily influenced by urbanization, influencing the nutritional and activity transitions (6).

Given its’ CVD burden, Africa faces several challenges including scarcity of high-quality data, financial constraints, competing priorities, limited skill sets, as well as diagnostic and management challenges (7). Recent and much needed increases in global and regional efforts to curb CVDs represent steps in the right direction and will need to be matched by surges in funding. The WHO Global Action Plan for the prevention and Control of NCDs has set targets for a 25% decrease in premature CVD mortality by 2025 and a third reduction for premature NCD mortality by 2030, covered by sustainable development goal (SDG) 3.4 (8, 9). Further, there are regional efforts such as Pan-African Society of Cardiology (PASCAR) goal to achieve a 25% reduction in hypertension prevalence in Africa (10). To date, modeling projections estimate a 48 and 52% increase for women and men in mean number of premature deaths due to CVDs by 2025 in SSA, provided continuation of current risk factor trends (11). Given the numerous constraints the continent faces, it is important to re-focus and invest in targeted interventions where the most gains can be made.

## Burden of cardiovascular disease in Africa

**FIGURE 1:**
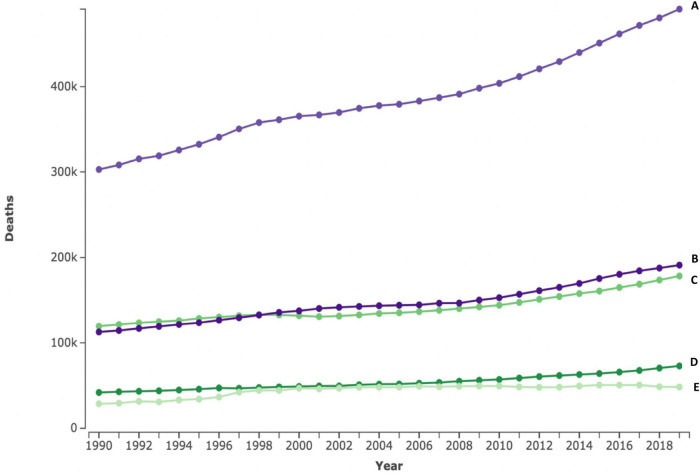
Absolute number of cardiovascular deaths in < 70 years for four SSA regions between 1990 and 2019. The top line represents the four Sub-Saharan African regions combined (A), Western (B), eastern (C), central (D), and southern (E) numbers in 100.000s. Figures extracted from the Institute for Health Metrics and Evaluation (IHME) (2).

CVDs are the leading cause of morbidity and mortality worldwide, contributing to about a third of all deaths (4). Globally, cardiovascular-related deaths have steadily increased by over a third from just over 12 million in 1990 to 18.6 million in 2019 (4). In Africa, CVDs are the largest contributor to the total NCD burden, accounting for 38.3% of NCD deaths and 22.9 million DALYs (5, 12). Contrary to several high-income countries (HIC) which recorded reductions in cardiovascular deaths (11), Africa has registered close to a 50% increase in the CVDs burden within the last three decades (2, 5). Aside from increases in major risk factors, this increase in CVD burden on the continent has been attributed to population growth and aging, demonstrated by a general downward trend in age-standardized mortality rates (4, 5, 11). While increases in mortality are seen in all SSA regions, the Western and Eastern regions have shown a steeper rise in CVD mortality (Figure 1, line B, C). These effects of population growth and aging on CVD incidence could considerably be mitigated by reducing the mean population blood pressure, with low-income countries (LIC) countries standing to gain the most benefits (13).

Drivers of cardiovascular mortality and morbidity in SSA are considerably different compared to HICs. Although ischemic heart disease (IHD) is identified as the leading cause of cardiovascular mortality globally (nearly 50% of all CVD deaths) (5), stroke (specifically hypertensive stroke), has historically been the greatest contributor to CVD mortality in Africa (12). Nevertheless, the rise in IHD is a major cause for concern (6), with recent modeling studies suggesting that IHD is responsible for the bulk of CVD burden on the continent (5). The THESUS study, a multicenter trial on heart failure (HF) in SSA, showed that the leading contributory causes to HF were hypertension, rheumatic heart disease (RHD), and cardiomyopathies (14). Similar results have been replicated in other centers (15).

Population-based studies in Africa demonstrate a high prevalence of cardiometabolic risk factors with 10-year absolute risk scores ranging 12.5–15.3% for men (16). Hypertension, diabetes and obesity were the most reported risk factors in a scoping review on current NCD research in SSA (17). Of concern, are the high rates of normal weight with central obesity associated with a higher risk of CVDs and mortality compared with high BMI without central obesity (18). Despite all these, Africa is still battling with endemic, and still neglected, CVDs such as RHD and endomyocardial fibrosis (EMF). Although declining, these diseases continue to contribute a substantial portion of the CVD burden in some of its regions (19, 20).

More than half of CVD deaths in Africa are categorized as premature mortalities, occurring between the ages of 30 and 70 years (5, 21, 22). The resulting large DALYs affecting the most productive age group culminates in serious social and economic consequences to the household, community, and nation at large (23). On average, Africa still has low health expenditure (averaged at 103 US$ per capita in 2016), with several countries still below the minimum recommended $44 per capita (8). This, combined with the lack of universal health coverage in most countries, necessitates high out-of-pocket costs for individuals, with resultant impoverishment and inequity in health care access (24).

## Risk factors for cardiovascular disease in Africa

**FIGURE 2:**
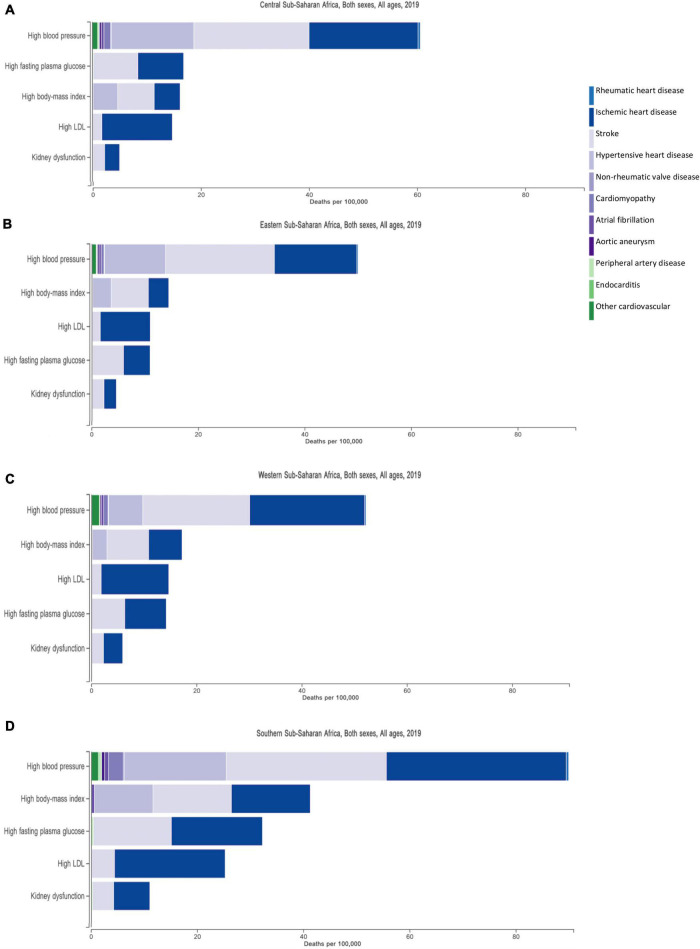
Metabolic risk factors and their contribution to the burden of cardiovascular disease in four regions in Africa, —Central **(A)**, Eastern **(B)**, Western **(C)**, and Southern **(D)** Sub-Saharan Africa. The three largest contribution of risk factors are illustrated by the three prominent bars making up ischemic heart disease, stroke and hypertensive heart disease.

The surge in CVD in SSA over the past decade is attributed to a rise in risk factors (11), largely driven by rapid urbanization (6). Four major cardiovascular risk factors—hypertension, high body mass index (BMI), high blood glucose and tobacco smoking- contribute to half of the global burden of disease (25). Hypertension is the leading contributor to stroke, ischemic, and hypertensive heart disease (HHD) burden. Figure 2 shows the burden of CVDs in four African regions and the metabolic risk factors attributing to these CVDs. The largest contribution of risk factors are seen for ischemic heart disease, stroke, and HHD, with high blood pressure having the highest share than any other risk factor, consistently across the four regions. The INTERHEART multicenter study further demonstrated the importance of nine potentially modifiable risk factors (including smoking, hypertension, diabetes, raised ApoB/ApoA1 ratio and abdominal obesity) that accounted for over 90% of all myocardial infarction (MI) risk (3).

The prevalence of CVD risk factors in Africa has been reported in several heterogeneous studies. The following paragraphs focus on selected classical risk factors with high potential to impact CVD burden in Africa in the foreseeable future (11).

### High blood pressure

Hypertension (HTN) is described as one of the leading health challenges on the African continent, with an estimated average prevalence of 27% (26). The reported prevalence of hypertension in Africa varies, with documented rates ranging from 15 to 70%, and a pooled prevalence of 30% in one systematic review (27). Although hypertension is a major problem in all regions of the continent, some regions have reported much higher prevalence. The ETHNA study, looking at hypertension in North African countries found a prevalence of 45.4% (28). However, higher figures have been reported in some populations in South Africa (SA), where hypertension estimates were over 50% (29). Even much higher figures documented in those > 50 years from the Study of Global Ageing and Adult Health (SAGE) SA data—and noted to be comparable to much older people in high income countries (16). On its own, hypertension accounts for a large population attributable risk (PAR), necessitating a special task force for sustained efforts toward its regional control by PASCAR (10, 11).

### High blood sugar

Twenty-four million people are estimated to be living with diabetes on the African continent (30), a 126% increase in both total and age-standardized DALYs between 1990 and 2017 (4). The risk of CVD increases with increasing fasting blood glucose (FBG) levels, even prior to levels sufficient for a diabetes diagnosis, at which point the risk of CVD increases 2–3 times (31). Rates of diabetes exhibit regional variations across the continent, with the highest prevalence recorded in North Africa (32). Within SSA, the southern region had double the burden of diabetes and kidney disease compared to other regions (4). Screening rates for diabetes are still poor with 50–60% of cases undiagnosed (30, 33). The current trends and potential burden of high blood sugar is a call for health systems on the continent to invest in programs that will enable population-wide prevention, early detection, and ultimately management of diabetes (32, 34). These efforts will be important to offset the enormous health system costs and catastrophic medical expenses associated with diabetes management including the high burden of associated vascular complications (34).

### Obesity

Obesity has been described as a “ticking time bomb” on the African continent, where rates continue to increase rapidly (35). Obesity, a result of energy imbalance between food consumed and energy spent in the form of physical activity, is uniquely more prevalent in women than men in Africa (30). According to WHO estimates, obesity rates range between 13.6 and 31% among 10 African countries with a high-obesity burden (36). High waist to hip ratio, an important predictor of CVD risk, is particularly problematic in some parts of the continent, reaching 61.6 and 73.4% for males and females (16). Obesity prevalence is highest in Northern (Egypt, Libya) and Southern Africa (South Africa, Namibia), with the lowest rates concentrated in the Saharan “central belt” (Ethiopia, Eritrea), amongst the poorest countries (36). Furthermore, variations exist in prevalence within settings, where higher rates are observed in urban compared to the rural populations (29), the result of urbanization and associated transitions.

### Tobacco smoking

Although the African region reported the lowest prevalence rates for tobacco smoking (estimated at 18.5%), the rate of decline is low relative to other regions (37). Several outliers exist, with four of the five global countries worldwide that were reported by WHO to have increases in tobacco use recorded in Africa (Egypt, Niger, Congo, and Lesotho), the fifth being Oman, in the Eastern Mediterranean region (37). It is worthwhile to note important data limitations with most of these countries is weak surveillance systems, presenting major problems with tobacco measurements (37). This has contributed to marked variations in the prevalence of tobacco smoking, overall ranging between 4 and 40% (38). Questions on tobacco use during survey data collection are new to many countries and lack of and/or poor data quality are documented major factors affecting WHO trends and country estimates (37). An important target under the United Nations’ Sustainable Development Goals (SDGs) relates to the control of tobacco smoking through global targets, that aims for a 30% relative reduction in prevalence of current tobacco use in those 15 years or older (39). The WHO Framework Convention of Tobacco Control (FCTC) outlines several policies important for achieving this target (38). Overall, 51 African countries have adopted several control strategies, making great strides, but many are lagging on several implementation measures such as tobacco tax excise duty (40). Raising the price of tobacco products through specific tax increases has been shown to be one of the most cost-effective policies to reduce smoking (41). For example, the MPOWER tobacco control measures advocated by WHO have resulted in reduction of initiation rates as well as increasing rates of stoppage in implementing countries (37). Despite this, Africa on average has the lowest tax to retail price ratio (41). Achieving these targets will require concerted efforts toward implementation of the control policies stipulated under FCTC that have worked well in other regions (42).

## Overview of cardiovascular pathologies in Africa

### Heart failure in Africa

Heart failure (HF) is a complex clinical syndrome resulting from a structural or functional impairment of ventricular function (43). It is the end result of a broad spectrum of cardiac pathologies, including genetic and systemic causes (44). Worldwide, the number of HF cases is over 64 million, collectively responsible for approximately 10 million years lived with disability (YLD) (44).

There are several factors that make HF particularly important in the African context. Foremost, acute HF is the leading cause of admission in specialized cardiology centers (14, 45). Secondly, HF in SSA commonly affects younger age groups, often presenting in late stages with an exceptionally high morbidity and mortality (15, 46). The rates of re-hospitalization and overall 1-year mortality are remarkably high, ranging between 22 and 58% (22, 45, 47). Thirdly, HF etiologies differ between high- and low- income countries (39, 48). While in HICs the main contributor to HF development is coronary heart disease, in SSA, the main contributors to HF are HHD, followed by cardiomyopathies and RHD, together accounting for three quarters of the causes of HF on the continent (15, 45).

**FIGURE 3:**
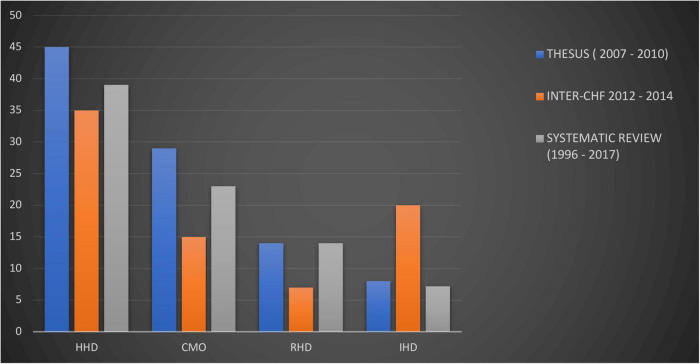
Trends in heart failure etiologies in Africa derived from heart failure cohorts from two different time spans (Thesus-HF and INTER-CHF Africa) and a systematic review (15, 45, 47). HHD, hypertensive heart disease; DCM, dilated cardiomyopathy; RHD, rheumatic heart disease; IHD, ischemic heart disease. CMO compiled as a combination of reported cardiomyopathies, idiopathic dilated, peri-partum, HIV-associated.

In a systemic review of heart failure in SSA, the contribution of IHD to heart failure etiologies on the continent was small, at 7.2% (45). Historically, the relatively low rates of IHD in the midst of abundant risk factors are thought to be explained in part by limited diagnostic capabilities for confirmation (49, 50). Nonetheless, there’s a rapidly rising trend in IHD within Africa and important regional differences should not be overlooked (6, 45). The INTER-CHF study recorded a substantial increase in HF resulting from IHD within five African countries (Figure 3). Important etiologies of HF which are more prevalent on the continent compared to other regions include infectious and inflammatory causes, such as tuberculous pericarditis (51), as well as nutritional deficiencies (52).

HF management in Africa is still challenged by lack of basic supportive investigations such as electrocardiograms, chest x-ray, and echocardiography, which are available in less than 50% of diagnosing centers (45, 53). The use of cardiac biomarkers and advanced invasive and non-invasive imaging is severely limited (53). Secondly, the use of evidence-based, guideline directed therapies such as beta-blockers and angiotensin receptor neprilysin inhibitors (ARNI), have been sub-optimal (15, 47). Thirdly, there is limited availability of device treatment for advanced heart failure, available only to the minority who can afford it (54).

The high rate of preventable heart failure should draw attention to prevention as an important area of focus for the continent. Other efforts should include investing in high quality epidemiological research -including therapeutic interventions, prioritizing early diagnosis, and optimizing treatments according to evidence-based medicine. Further, innovative strategies such as tech-enabled ways to enhance patient follow-up and promote adherence may lead to improved outcomes.

### Atherosclerotic cardiovascular diseases

Sub-Saharan Africa has witnessed a substantial increase of atherosclerotic cardiovascular diseases (ASCVD) over the last three decades. According to the Global Burden of Disease (GBD) estimates, the highest contribution to CVD burden on the continent is attributed to atherosclerotic diseases, with 71.4, 37.7, and 154% increases in the burden (all age DALYs) of ischemic heart disease, stroke and peripheral artery disease since 1990 (5) (Table 1). While the figures from the GBD may be an overestimate due to imprecise data from poor country registration and surveillance leading to a reliance and limited data for statistical modeling tools, verbal autopsy and hospital data are also beginning to support the growing contribution of atherosclerotic disease to the CVD burden (55, 56). Hypertension is the most common risk factor driving this burden which, combined with other highly prevalent and poorly treated risk factors such as diabetes, obesity and dyslipidemia, are responsible for the increasing ASCVD burden (11, 57).

**TABLE 1:** All-age total DALYs in 1990 and 2017 and percentage change from 1990 to 2017 from CVD in sub-Saharan Africa [GBD].

	1990 (′000)	2017 (′000)	% Change 1990–2017
**Cardiovascular diseases**	**15 565.2 (14 490.3–16 567.9)**	**22 860.8 (21 507.2–24 304.8)**	**46.9%**
Rheumatic heart disease	973.2 (822.8–1129.2)	1036.7 (866.3–1228.8)	6.5%
Ischemic heart disease	4928.7 (4483.3–5383.4)	8449.7 (7813.7–9248.5)	71.4%
Stroke	5904.4 (5431.4–6399.4)	8129.9 (7579.9–8673.7)	37.7%
Hypertensive heart disease	1057.5 (701.9–1352.7)	1597.5 (1033.8–2056.3)	51.1%
Non-rheumatic valvular heart disease	99.2 (80.8–127.7)	162.2 (141.9–188.5)	38.9%
Cardiomyopathy and myocarditis	818.6 (645.5–987.3)	994.0 (867.2–1118.5)	21.4%
Atrial fibrillation and flutter	113.8 (92.5–138.4)	234.8 (190.3–283.8)	106.4%
Aortic aneurysm	136.6 (102.5–175.7)	184.4 (151.5–213.1)	35.0%
Peripheral vascular disease	25.8 (15.9–38.9)	65.5 (41.7–91.0)	154.0%
Endocarditis	401.6 (276.9–579.4)	446.2 (359.7–548.7)	11.1%
Other cardiovascular and circulatory diseases	1105.8 (851.0–1638.7)	1559.9 (1238.4–2200.0)	41.1%

Age-standardized and all-age DALY rates and total DALYs in 1990 and 2017, and percentage change from 1990 to 2017. Gouda et al. (5). [cited 2022/05/26]; p.e1379. Available from: https://www.thelancet.com/action/showFullTableHTML?isHtml=true&tableId=tbl1&pii=S2214-109X%2819%2930374-2.

#### Coronary artery disease

Coronary artery disease (CAD) is the leading cause of death globally, responsible for over 50% of all cardiovascular deaths (58). Over the past three decades, there has been a substantial increase in CAD prevalence, previously responsible for only 6% of CVDs in the early 1990’s (59). Current estimates suggest IHD is responsible for close to 40% of all age- total cardiovascular DALYs (5). CAD in Africa affects the younger, more productive population and therefore has substantial impact on the economy of African nations (60). In the INTERHEART study, African patients presenting to hospital with acute coronary syndrome were significantly younger than those from the rest of the world (52 ± 12 years vs. 57 ± 12 years, respectively), and had higher levels of hypertension, diabetes, smoking, and depression (57).

African countries face several challenges with regards to CAD. Apart from poor recognition of angina symptoms (61), there is sub-optimal diagnosis and treatment of CAD and its risk factors. Lack of access to and variable quality of essential medications to treat CAD also compounds the burden (62). Secondly, delayed hospital presentation after symptom onset hampers life-saving emergency care (10, 63, 64). Thirdly, given limited access to fibrinolytic therapies and barely existent percutaneous coronary intervention services, emergent coronary revascularization remains extremely challenging and unaffordable to many. Together, this translates into high mortality for patients presenting to health units in the region (65).

#### Stroke

As with CAD, there has been a substantial decline in global, age-standardized stroke mortality rates over the last two decades, possibly as a result of improved stroke care. However, this decline in incidence has not been reflected in Africa (66). The annual incidence rate of stroke in Africa, 316 per 100,000 individuals, is striking. Africa also records the highest case-fatality rates for stroke at 30 days in the world, ranging from 16.2 to 46% (67).

Inadequate control of stroke risk factors, poor recognition of the urgency of symptoms leading to delayed presentation, lack of timely diagnosis and fibrinolytic services, and the dearth of post-stroke rehabilitation services are major drivers of stroke burden in SSA (67, 68). It is also possible that the difference in stroke sub-types in Africa contributes to this burden, where a relatively larger fraction of intracerebral hemorrhage occurs in the younger population and portends worse outcomes (68). Within the atherosclerotic stroke subtypes, there is a considerable proportion of small vessel disease even among younger age groups, highlighting poor risk control and perhaps suggesting that vascular dementia may be particularly troublesome in the region (69).

#### Improving outcome for atherosclerotic cardiovascular disease in Africa

Moving forward, investment in cost-effective ways for the prevention and treatment of risk factors for atherosclerotic disease is paramount. Systematic screening, improving access to medication, and implementing standardized guideline-directed clinical management may prove vital to control efforts (8). Investing in population-level interventions such as promoting physical activity, reducing levels of salt intake, and increasing fruit and vegetable intake through policies in different sectors would have wider impacts in reducing risk and disease burden (10, 70). Further financing emergency care for these conditions is needed, integrating pre-hospital emergency services with the rest of the health system to better ensure linkage with specialized centers. Early fibrinolysis is worth developing for both acute coronary syndrome and acute stroke, given the paucity of catheterization laboratories for emergent endovascular procedures. Finally, there is evidence that the development of multi-disciplinary specialized care centers to manage post-MI and stroke patients leads to improved outcomes (71). Multi-disciplinary specialized care centers have been successfully implemented in some parts of the African region (72) and it is important that such efforts are scaled up throughout the continent.

### Hypertension

Hypertension is the number one driver of CVD in Africa. According to recent estimates, the African region has the highest prevalence of hypertension in the world (26), as well as the greatest rise in prevalence since 1990 (4). Africa also has the highest age standardized DALYs secondary to HHD, particularly central SSA (4). Behavioral risk factors as a result of urbanization, aging, social stress, and poor access to health care have been the main drivers (11).

Dubbed the “silent killer,” a result of the initial asymptomatic nature of the disease, many present for the first time to health care facilities with fatal complications. Hypertension is responsible for more than half of total cardiovascular related deaths, being the main driver of IHD, HHD, and stroke burden on the continent (2, 11; Figure 2). In a 3-year prospective study in Tanzania, hypertension-related deaths accounted for an in-hospital mortality of 20% (73). These were largely attributed to hypertensive stroke (53.2%), followed by hypertensive heart failure (27.1%), hypertensive emergencies (17.5%), and hypertensive renal disease (2.2%) (73).

**FIGURE 4:**
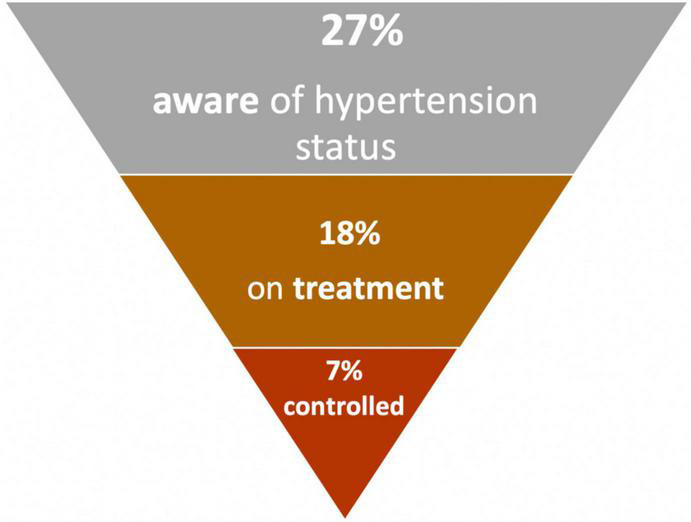
Prevalence, awareness, treatment and control of hypertension in Africa (27).

The benefits of adequately controlled blood pressure are well-known, with reported reductions in cardiovascular mortality as large as 38.0 and 30.4% among females and males (74). Of critical importance is the large burden of undiagnosed, untreated, and uncontrolled hypertension (27). A pooled analysis of data from SSA revealed that only 27% of those with hypertension were aware of their status, and just 18% of those with hypertension were receiving treatment (27). Even among treated cases, poor control is common (10). Figure 4 depicts this cascade of hypertension from prevalence, awareness, diagnosis, and control (27–18–7%), demonstrating that a very small percentage of the total population is actually treated and controlled (27). Important to note that there was significant heterogeneity in these parameters across the studies pooled, mostly explained by variations in the mean age of participants and study designs (27).

The need for hypertension control in Africa is undoubtedly one of the highest priorities. Integrating care widely in primary health facilities by equipping them with tools and quality, accessible medications to manage hypertension in concert with other cardiovascular risk factors should be a priority (75). One approach would be to leverage the existent and widespread HIV care network. Employing task-shifting to provide guideline directed lifestyle and pharmacological management, whilst gearing efforts toward universal health coverage are approaches that may prove beneficial (76). The HEARTS (Healthy-lifestyle counseling, Evidence-based treatment protocols, Access to essential medicines and technology, Risk-based CVD management, Team-based care, and Systems for monitoring) technical package is an example of a scalable strategy to improve HTN control at the primary care level, adopted by a few countries on the continent (77). Likewise, population-wide interventions for addressing behavioral risk factors such as high salt intake, physical inactivity, cigarette smoking, and poor dietary habits should be active on the policy agenda. In modeling studies, salt reduction contributed to at least 15% of the reduction seen in the probability of dying from CVDs (13). Policies targeted at whole populations such as mandatory reformulation will be important to achieve significant reductions in salt consumption among other interventions (78). The strides to successful implementation of salt reduction policies are well demonstrated by the South Africa’s mandatory salt reduction policy that recorded a reduction in salt intake of 1.2 g/day within 2 years of implementation (79). Results and follow-up from large clinical trials in the future will be important informants on whether this translates into reduction of disease burden and the extent thereof.

### Human immunodeficiency virus and cardiovascular disease morbidity in sub-Saharan Africa

Sub-Saharan Africa (SSA) has the highest burden of HIV in the world, accounting for more than two thirds of the global HIV burden (3). Remarkable strides have been gained in managing chronic HIV in SSA–with increased access to anti-retroviral therapy (ART), viral suppression has resulted in less HIV-defining illnesses and a rise in non-AIDS defining illnesses. The drivers are multi-factorial, however, chronic HIV infection is a well-recognized risk factor for CVD, including myocardial infarction (80), heart failure (81), and stroke (82). SSA accounts for half of the global burden of DALYs lost due to HIV-related CVD, and the PAR for HIV-associated CVD reaches 15% in parts of the continent (83).

In high-income countries, people living with HIV (PLHIV) and hypertension have higher risks of cardiovascular events and all-cause mortality compared to HIV-uninfected persons with hypertension or PLHIV with normal blood pressure (84). Absolute differences in risk may be magnified in SSA because of poor access to hypertension care and treatment. In addition, the cardiovascular risk prediction model used in the general population have not proved accurate in predicting CVD risk related to HIV (85). Despite the high prevalence of traditional risk factors amongst a Ugandan population with HIV, a low prevalence of coronary calcium score was recorded in this population (86). Research is needed to highlight the parameters that better predict risk in PLHIV. The ongoing Ndlovu study comparing a cohort of HIV negative, and HIV positive patients will give further insights on risk prediction (87).

Integrating CVD care into HIV programs is key to mitigating the growing burden of CVD in HIV populations in Africa. As an example, PLHIV attending primary health care facilities in rural South Africa received better care for both hypertension and diabetes (88). But in general, many HIV programs in Africa are not keen on screening for CVDs and their risk factors, which delays early detection and institution of appropriate measures to slow disease progression (89, 90). The “double care” imposed on HIV/CVD patients translates into increased expenditure and worse outcomes as limitations in infrastructure compromise optimum, holistic patient care. There is need to look beyond only the HIV-set program targets to overall patient wellbeing (91, 92). As a continent, several lessons can be learned on integration of HIV and CVD care, from existing programs in countries such as South Africa, Malawi, Kenya and Swaziland (93). Opportunities exist for developing national health policies that recognize the need for care integration, as well as efficient coordination between policymakers, researchers and implementers in leveraging on successful HIV programs (93).

### Cardiomyopathies

Cardiomyopathies are defined as disorders in which the heart muscle is structurally and functionally abnormal, in the absence of CAD, hypertension, valvular disease, and congenital heart disease (CHD) sufficient to cause the observed myocardial abnormality (94). They are classified as dilated cardiomyopathy (DCM), hypertrophic cardiomyopathy (HCM), restrictive cardiomyopathy (RCM), arrhythmogenic right ventricular cardiomyopathy (ARVC), and unclassified cardiomyopathies. All cardiomyopathy phenotypes have been reported in the African population with significant contribution (20–30%) to heart failure in adults (95). In particular, DCM and endomyocardial fibrosis (EMF) are endemic in SSA (96).

#### Dilated cardiomyopathy

DCM is defined by the presence of left ventricular dilatation and left ventricular systolic dysfunction in the absence of abnormal loading conditions (hypertension, valve disease) or coronary artery disease sufficient to cause global systolic impairment (94). DCM is the most prevalent of cardiomyopathies and one of the commonest causes of heart failure in black Africans, only second to hypertension (97). Over the past 50 years, the prevalence of DCM in some regions of the African continent has greatly increased from 10 to 17% (96) to approximately 35% of all cardiac diseases (98). An ongoing systematic review on risk factors and prevalence of DCM in SSA will give an insight on the possible factors underlying these trends (99).

Two thirds of patients with DCM have poor prognostic factors and die within 5 years of their first symptoms (100). The causes for DCM are largely unknown, hence commonly referred to as idiopathic and generally accepted that the disease probably represents a final common expression of myocardial damage that could be provoked by multiple insults. The causative factors that have been examined in Africans include infections and myocarditis, autoimmune mechanisms, iron overload and other metabolic factors, genetic factors, alcohol and nutritional deficiencies, chemotherapy, and pregnancy (96).

#### Other cardiomyopathies

A number of other cardiomyopathies contribute to CVDs in Africa, albeit much less common than DCM. Hypertrophic cardiomyopathy (HCM) was hardly diagnosed in Africa prior to widespread availability of echocardiography (97). Current reports for HCM have documented a prevalence of 0.2–2% (97, 101, 102), with some reports suggesting a higher prevalence in people of African descent compared to Caucasians (103). Limited availability of intra-cardiac device (ICD) implantation and other invasive treatment ultimately have influenced the poor outcomes of patients with HCM in Africa.

Endomyocardial fibrosis (EMF), an endemic cardiomyopathy, is believed to be the most common primary form of restrictive cardiomyopathy in Africa, elaborated on later in this review. Other rare forms of cardiomyopathies which have been reported in Africa include ARVC and NCLV (non-compaction of the left ventricle). The diagnosis of ARVC is still a challenge in SSA countries. The only available data on this condition comes from a single center study in South Africa, where 12 cases were reported in 6 years (104). NCLV is characterized by prominent left ventricular trabeculae and deep inter-trabecular recesses (94). It is not as rare as once believed, with documented reports from different African countries since 2006 (105).

Overall, investments into further research will be informative in enhancing our understanding of the etiology and management of cardiomyopathies in Africa. Lack of confirmatory diagnostic facilities, i.e., endomyocardial biopsy, cardiac magnetic resonance imaging, and genetic studies have contributed to under-diagnosis of some cardiomyopathies in Africa; however, the rising use of echocardiography has increased suspicion and may result in increased reporting in the coming years.

### Rheumatic fever and rheumatic heart disease

RHD results from recurrent episodes of acute rheumatic fever (ARF), a long-term complication of Group A streptococcal (GAS) infection. ARF is thought to be an autoimmune-mediated response to interactions between the body and certain components of the GAS bacterial cell wall protein. RHD is currently estimated to affect approximately forty million people worldwide, the majority of whom are children and young adults in sub-Saharan Africa (4). About 395,000 children are thought to die from RHD annually, largely contributing to premature CVD mortality, with an average age at death of 28 years (106). Women of childbearing age suffer the highest burden of RHD (106, 107).

A combination of factors including health system factors, poor health seeking behavior, and evolving virulence of the causative agent have resulted in the persistence of GAS and its long term sequelae, RHD, in SSA (107). The burden of RHD is further driven by poor recognition, diagnosis and treatment of acute GAS infection and missed opportunities to identify RHD in its early, asymptomatic stage (latent RHD). Consequently, most cases present to hospitals with severe valve disease either requiring expensive surgery that is often not accessible to most, or when the benefit-risk ratio from surgery is unfavorable.

Given its long and extended life cycle, RHD does offer several opportunities for life saving interventions. *Primordial prevention* involves a multi-pronged approach at the government level that targets improvements in living conditions of the population including improving housing standards to prevent overcrowding, fostering good hygiene practices, and promoting education. *Primary prevention* aims at preventing the rheumatic process from starting and targets prompt detection, treatment, and follow up of GAS pharyngitis. Although several interventions have been implemented in Cuba and Nicaragua, this approach requires near overhauling of the health system and may not be cost effective (108). *Secondary prevention* that targets reducing recurrent episodes of ARF and thus RHD progression is by far the most effective intervention to date. A registry-based approach based on monthly injectable benzathine penicillin (BPG) has been shown to be both effective and affordable (108). *Tertiary prevention* through catheter-based therapies and valve surgery programs is still a much-needed part of RHD case management in SSA, but insufficient access and associated financial constraints of these therapies leave this as an area needing further investment in many countries.

Important questions remain regarding RHD: (1) How can health systems in LMIC improve access to primary prevention services? (2) In patients with ARF or RHD, which delivery systems can help improve adherence to BPG medication? Are depot, long term preparations of penicillin feasible? (3) Does ARF present differently in Africa and are health care workers adequately equipped to diagnose, manage and perform its surveillance? And (4) What are the immunological processes that underlie progression from ARF to clinical RHD? Several important research works are on-going examining some of the aforementioned aspects of RHD management. The landmark GOAL trial showed that BPG prevented progression of latent RHD in children who were administered monthly BPG (109). Further, studies are looking at GAS molecular biology and ARF immunology using omics technology which could shed light on our understanding of the disease continuum and future pathways for RHD elimination.

### Cardiac arrhythmias

The CVD epidemic in Africa includes and/or is a harbinger for cardiac arrhythmias and their related health impact (110). The spectrum of arrhythmias in SSA has been recently described in systematic reviews (111, 112), however, the available data still remains quite limited (113). Overall, arrhythmias remain a neglected field of cardiology in Africa (114).

Chief among the cardiac arrhythmia maladies is atrial fibrillation (AF), noted to have a higher prevalence in the general population than previously thought (104). A recent systematic review has summarized the epidemiology of AF in Africa where the prevalence ranged between 6.7 and 34.8% in patients with ischemic stroke, 9.5 and 46.8% in those with RHD, and between 5 and 31.5% in patients with dilated cardiomyopathy (115). The main risk factors for AF were hypertension, valvular heart disease, and cardiomyopathy. Complications of AF included heart failure in about two thirds and stroke in 10–15% of cases (115). The use of anticoagulation for stroke prevention is challenging, given its use is inconsistent and largely suboptimal (115). Overall, the management of AF is associated with exorbitant costs, contributing to the challenges (115, 116).

Likewise, data on the epidemiology and management of supraventricular arrhythmias, ventricular arrhythmias, brady-arrhythmias and sudden cardiac arrest in Africa remain scant (111). The Africa Heart Rhythm Association (AFHRA) (117) has described most of what we know about the epidemiology and access to care of cardiac arrhythmias in Africa (112, 114). Major gaps described include the low rates of cardiac ICD insertions and rarity of invasive arrhythmia treatment centers in SSA (54, 111).

Overall, there is a growing need for appropriate arrhythmia care in Africa, an area that remains largely unmet because of complex multi-level challenges (112). Locally relevant solutions, such as “warfarin care bundles” designed to address anticoagulation challenges have been put forward (118). Since its inception, AFHRA is taking leadership in expanding cardiac arrhythmia research and increasing access to care. Multifaceted strategies have been implemented, with particular emphasis on personnel training through fellowship programs (119) and focusing on preventive care (112, 114). For cardiac pacing, the use of reconditioned pacemakers might be a short-term solution to the cost-related challenges that limit access to device therapy in Africa (120).

### Neglected diseases specific to Africa: Endomyocardial fibrosis

**FIGURE 5:**
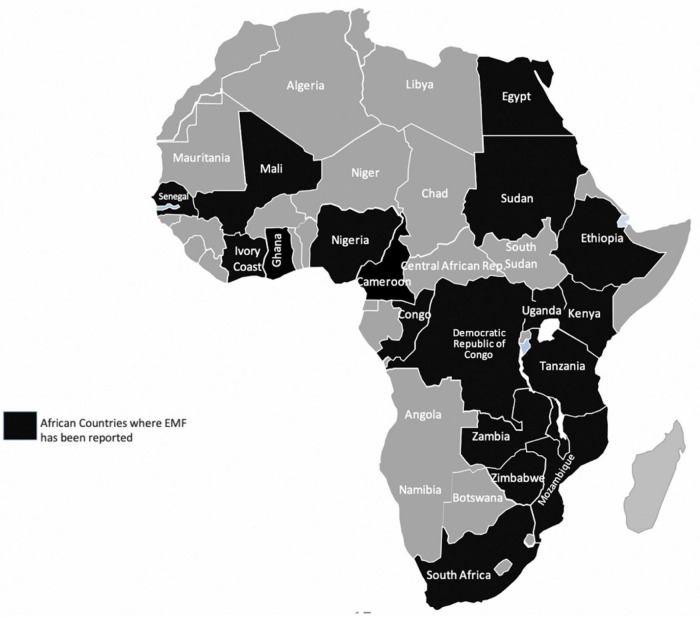
Countries in Africa where Endomyocardial fibrosis (EMF) has been described previously. Data extracted from Mocumbi et al. (126).

Endomyocardial fibrosis (EMF) is the most common form of restrictive cardiomyopathy globally (121). Since its discovery in Uganda in 1938, EMF has been described in several parts of the world with majority of cases clustered in the tropical regions of Africa, Southeast Asia, and Latin America. Nearly half of the published cases globally originate from SSA (122–126; Figure 5). In endemic areas, EMF largely affects children and young adults living in conditions of poverty and contributes to premature cardiovascular morbidity and mortality (124).

The etiopathogenetic mechanisms underlying the disease still remain debatable, in part due to lack of investment in research. Environmental (hyperesinophilia from helminths and other tropical infections, geochemical toxins and diet), socioeconomic, and genetic factors are all thought to play a role (122, 125–127). Focal or diffuse endocardial thickening resulting from fibro-collagen tissue deposition, typically involving the ventricular apices and atrioventricular (AV) valves, is characteristic of the disease and this results in varying degrees of AV valve regurgitation and subsequent heart failure. Both acute and chronic phases of the disease are recognized (122, 128).

Advanced EMF carries a poor prognosis. Medical therapy is directed toward treatment of heart failure symptoms. Death often results from refractory heart failure, arrhythmias and thromboembolism. Corrective surgery, which has been shown to improve survival and quality of life (122, 129), is virtually inaccessible for the vast majority of EMF patients in Africa. Recent reports suggest a decline in EMF prevalence in once endemic areas in Africa (130–132) and India (133), most likely related to improvement in socioeconomic conditions. These declines likely represent reduction of symptomatic cases, and it’s unclear whether asymptomatic disease still persists in previously endemic communities. Epidemiologic studies to assess community burden of EMF in Africa have only been undertaken in Mozambique (52, 134). Recently, echocardiography-based EMF severity staging criteria have been proposed but these are yet to be validated (52). More targeted community screening for EMF to understand the true burden of asymptomatic EMF in Africa and prioritize treatment pathways and surgery for those affected is needed.

### Cardiovascular disease in pregnancy

In patients with diagnosed and undiagnosed CVD, pregnancy and labor pose remarkable physiological and hemodynamic stress and portends significant negative consequences on both maternal and fetal outcomes. Globally, 1–4% of pregnancies are complicated by maternal CVDs (excluding hypertension) (135). On its own, hypertension occurs in 10% of all pregnancies, while pre-eclampsia complicates 2–8% of all pregnancies and is responsible for the highest maternal mortality (136, 137).

A systematic review on antenatal heart disease burden in South Africa showed a prevalence of 0.6% (138). Whereas the pattern of CVD in pregnancy in developed countries consists mostly of congenital heart disease, the spectrum of antenatal heart disease in Africa is dominated by RHD (accounting for 88–90%, and predominantly of mitral valve disease), followed by cardiomyopathies and congenital heart diseases (138). Mitral stenosis, prosthetic valves, and cardiomyopathies portend poor outcomes (138, 139). Peripartum cardiomyopathy (PPCMP) has been reported to affect 17.4% of mothers presenting with heart failure in the peripartum period and carries a high maternal mortality (140). Marked differences exists in the prevalence of PPCMP across the continent; whereas 1 in 96 deliveries were reported in Nigeria, the prevalence of 1 in 1,000 deliveries reported in South Africa was more than ten times less (141, 142).

Challenges still exist in the management of patients with heart disease in pregnancy. African women in general are under social pressure to bear children and will take the risk despite the severity of disease (143). Secondly, there is limited awareness and access to preconception counseling, with many seeking healthcare after or at the end of the first trimester whilst on contraindicated chronic cardiac medications and warfarin anticoagulation (144). Moreover, there is still limited access to surgical procedures for most women in the childbearing age (145). Finally, anticoagulation for AF, often accompanying mitral stenosis, is another entity that is even more challenging in pregnancy in addition to general challenges discussed above. Monitoring of warfarin is difficult, while heparin is less affordable (146).

Improving outcomes of pregnant mothers with CVD requires an integrated and context- specific approach that engages the patient (understanding the patient needs), and involves a multi-disciplinary team headed by an obstetrician and cardiologist. Joint obstetric-cardiac care has been shown to improve both maternal and fetal outcomes (147). Sustained efforts, including resources and advocacy for RHD screening to allow early diagnosis and timely interventions could improve outcomes.

### Congenital heart disease

Congenital heart disease accounts for one third of all birth defects (148). The global birth prevalence rates of CHD show significant geographic variation, with African rates of 1.9–2.3 per 1,000 births being much lower than the global rates of 9.1–9.4 per 1,000 births (148, 149). The lower prevalence rates and the under-representation of critical CHD phenotypes such as ductal-dependent lesions seen in Africa is generally thought to be an under-estimation due to lack of accurate epidemiological data and reduced survival (149–152). Dramatic advances in diagnostic and treatment options have seen 85% of all children born with CHD in high-income countries now survive into childhood (153). This includes 80% survival for complex lesions, such as truncus arteriosus and transposition of great arteries, and 95% of those with simple defects, like ventricular septal defects, likely reaching adulthood (153–155). Unfortunately, children in Africa generally have worse prognosis. Whilst there was a global decline in mortality from CHD, SSA recorded a tremendous increase in mortality between the years 1990–2017 (150). The southern region of SSA was the only region that registered a decline in CHD mortality rates (150).

Several challenges affect CHD care in Africa: (1) prenatal diagnosis of CHD in Africa is rare because of severely limited antenatal screening capacity for CHD (156, 157). (2) Late presentation with cardiac and pulmonary complications is the norm (157, 158). (3) Access to definitive surgical or transcatheter treatment for CHD is severely limited across the African continent with 90% of children with CHD having no access to appropriate surgical care (159, 160), leading to a considerable number of deaths in the neonatal and infancy period (161). (4) Although early surgical outcomes are acceptable (162, 163), long-term follow up data from the continent is poorly described, and (5) The genetic basis for CHD in Africa is still grossly understudied, and the impact of environmental factors are not fully explored (164). Ongoing research such as the PROTEA project by researchers from the University of Cape Town hopes to create an accurately phenotyped and genotyped longitudinal CHD cohort in southern Africa (165).

To improve CHD care, it is important to: (1) Increase funding to strengthen already existing CHD centers through both governmental and non-governmental organizations, (2) support training and mentorship of super-specialists across the CHD spectrum, and (3) increase awareness, promote early neonatal screening, and treatment through universal health insurance.

## Africa’s progress toward sustainable development goal 3.4—Cardiovascular disease component

**FIGURE 6:**
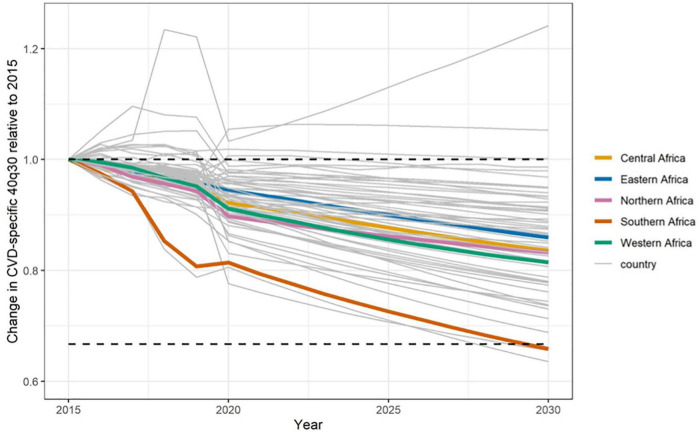
*Status quo* projections for 40q30 through 2030 for cardiovascular disease in Africa by country and 5 sub-regions (Southern, Central, Eastern, Western and North Africa). The horizontal lines denote baseline 40q30 in 2015 and a third reduction in 40q30 with respect to 2015 baseline rate (13).

Currently half way between 2015 and 2030, *status quo* projections indicate that Africa’s overall progress is insufficient to achieve SDG 3.4 by 2030 (166). Data from the years 2010–2016 revealed stagnation in the probability of dying prematurely from four major NCDs, including CVDs in many countries in Africa. At best case scenario, these countries will have to achieve rates of decline as good as the ten best performing countries worldwide—an ambitious target given Africa’s current projections (166). Figure 6 shows the current projections to 2030 in African countries on their progress toward cardiovascular premature mortality reductions from 2015, the baseline year (13). These projections begin from the year 2020. For the majority of countries, the rate of decline in the probability of dying prematurely between 30 and 70 years of age (40q30), has been too slow to reach the 33% reduction target. Many are projected to attain between 10 and 20% reduction. The two lines well above y = 1 (baseline) are Cape Verde (which increased from 6.3% in 2015 to 7.9% in 2030) and Eritrea (which increased from 13% in 2015 to 13.9% in 2030). Comoros and Equatorial Guinea both hover around no change from 2015 to 2030. Conversely, South Africa and Algeria are on track to achieve the target with SA projected to see the largest decrease (from 8.9% in 2015 to 5.6% in 2030, representing a 36.5% relative reduction) and Algeria projected to achieve the 33% relative reduction (where the 40q30 decreased from 9.9% in 2015 to 6.5% in 2030). Below we summarize current gaps and highlight important priorities going forward for the continent.

## Important gaps in cardiovascular health in Africa

• There’s inadequate research productivity from Africa which contributes to the widening gap in health outcomes with other countries (167, 168). Importantly, lack of systematic surveillance data covering vital statistics such as causes of mortality within countries hampers disease burden estimates and consequently affects monitoring and evaluation of interventions.
• Data on the physician-to-population ratio confirm a persistent human resource crisis in the health sector, which is even more pronounced for sub-specialties (24). Overtime, an increase in medical schools has been marred by faculty shortages and likely reduced quality, poor renumeration, and consequently “brain drain” to other countries (169).
• There is a large gap to fill for general access to CVD care, contributory factors include:
  ∘ Low health budget allocation—According to the WHO, most African countries have insufficient health financing systems with high out of pocket expenditures. Almost half of all African countries still have out of pocket expenditures of above 40% (170). In general, health spending in still very low in Africa, with the Central, Western and Eastern African regions documented to have the lowest worldwide (171). Moreover, there is a huge reliance on donor funding among several African countries and relatively less government health service expenditure on primary care (171).
  ∘ Lack of universal health insurance and consequently high out-of-pocket expenditure, sometimes resulting in catastrophic health spending.
  ∘ Inadequate access and long-term affordability of evidenced-based CVD medications, including important drugs for CVD prevention (172).
  ∘ Inadequate facilities for advanced cardiovascular imaging and procedures (54).
• Low education and health literacy levels (173), affects health choices made, including health-seeking behavior (174) partly contributing to challenges described such as the late presentation of CVDs and subsequently poor prognosis. In terms of risk factors, those with more years of schooling are less likely to be involved in risk behaviors such as excessive drinking and smoking (175). Finally, there is a paucity of cost-effective, integrated and evidenced-based approaches for CVD prevention targeted at whole populations (11) and supported by effective policies and government commitment.

## Key priorities

• *Prevention*: identifying and implementing cost effective, preventive strategies targeting both high risk and the general population is the most reliable solution to the rising burden of CVD in Africa. Preventive efforts such as the adoption of the FCTC for tobacco control (176), introducing sugar tax for processed foods (177), and implementing population-based interventions that promote healthy lifestyles have proven effective in several settings. This will require sustained advocacy by in-country expert groups of public health specialists and other policy makers to prioritize these policies in order to improve CVD outcomes.

Hypertension Prevention deserves concerted efforts given its contribution to CVD in Africa. Universal and periodic screening, diagnosis and guideline-directed treatment through strengthening and integration into primary care facilities, task-shifting, and universal availability of effective drugs should be prioritized in most countries. Intensively accelerating control through two population-based interventions—pharmacological guideline-directed treatment of hypertension and “upstream” policies targeted at a reduction in salt consumption could reduce CVD mortality by up to 22% relative to *status quo* (13). The WHO STEPwise surveillance tool for in-country monitoring of HTN detection and control has been recommended by PASCAR (9).

• *Increase Research Outputs*: high-quality epidemiological and implementation research, as well as dedicated investments toward strengthening vital statistic data and systematic surveillance, will inform practice and contribute towards identifying and evaluating’ sustainable interventions in various settings (70), track progress, and strengthen policy development and adoption.
• *Improve Training*: priorities to improve training should include establishing in-country cardiology training centers and strengthening existing training centers through bench marking and fostering collaboration with strong external cardiology training institutions.
• *Improve Access*: a number of options exists to increase access to cardiovascular care:
  ∘ reducing out-of-pocket expenditure and implementing health financing plans with the objective of increasing equitable and effective access to CVD care (169). Among other factors, health reforms to promote progress towards UHC will involve country commitments to increasing revenue generation and overall government expenditure on health, employing a whole system approach (169). Increasing government expenditure on health by allocating at least 15% of their national budgets to the health sector, including a minimum of US$ 44 per capita for health funding (170).
  ∘ task shifting of preventive cardiovascular care strategies like screening, lifestyle modification counseling, routine, uncomplicated, follow-ups, specialized care linkage, and coordination of NCD support groups (76).
  ∘ investing in infrastructure for high-quality care including acute cardiovascular care is an essential component of reducing cardiovascular mortality (71).
  ∘ incentives for health care workers that aim to enhance service delivery and retain knowledgeable and skilled medical personnel within countries (170).

## Conclusion

The burden of CVD in Africa is a result of increasing and unchecked risk factors with considerable heterogeneity in risk trends across its regions, that has led to explosive rates of CVD-related morbidity and mortality on the continent. Despite the sustained efforts to combat CVDs on the continent by the different advocacy groups such as WHO, PASCAR, and World Heart Federation, the adoption of effective policies is lagging in many African states. This review has highlighted several gaps in various sectors of CVD care on the continent. Overall, the evidence suggests applying preventive strategies to whole populations that target major risk factors will make important contributions toward reaching set targets by 2030. Specifically, for Africa, intensified and tailored regional efforts need to be channeled to lower blood pressure and other risk factors that should be coupled with continued surveillance and reliable data collection and monitoring programs. Concerted efforts from global, regional, and local experts are needed to increase advocacy for CVDs. Efforts should focus on identifying innovative ways to improve access and service provision at a primary health care level, investing toward universal health coverage, investing in cost-effective preventive approaches that are easily adaptable, and prioritizing research. Given country commitments to strengthen health systems, adopt and implement key metrics targeting awareness, prevention and management of CVDs, significant strides are possible toward changing the current trajectory of CVD burden in Africa.

## Author contributions

EO, JR, DN, and NM contributed to conception and initial outline of the review. NM, DN, TA, WZ, IS, JN, WA, SL, EN, JR, EO, and JK contributed to writing different subsections. JK, EO, EN, SL, DN, and NM contributed to manuscript content and language editing. All authors contributed to manuscript revision, read, and approved the submitted version.
